# Activated T cells sustain myeloid-derived suppressor cell-mediated immune suppression

**DOI:** 10.18632/oncotarget.6662

**Published:** 2015-12-18

**Authors:** Laura Pinton, Samantha Solito, Vera Damuzzo, Samuela Francescato, Assunta Pozzuoli, Antonio Berizzi, Simone Mocellin, Carlo Riccardo Rossi, Vincenzo Bronte, Susanna Mandruzzato

**Affiliations:** ^1^ Oncology and Immunology Section, Department of Surgery, Oncology and Gastroenterology, University of Padova, Padova, Italy; ^2^ Clinic of Pediatric Hemato-Oncology, Department of Women's and Children's Health, University of Padova, Padova, Italy; ^3^ Surgery Section, Department of Surgery, Oncology and Gastroenterology, University of Padova, Padova, Italy; ^4^ Surgical Oncology Unit, Veneto Institute of Oncology - IOV-IRCSS, Padua, Italy; ^5^ University of Verona, Department of Pathology and Diagnostic, Section of Immunology, Verona, Italy; ^6^ Veneto Institute of Oncology - IOV-IRCSS, Padua, Italy

**Keywords:** MDSC, immune suppression, tolerance, immune regulatory pathways, tumor microenvironment, Immunology and Microbiology Section, Immune response, Immunity

## Abstract

The expansion of myeloid derived suppressor cells (MDSCs), a suppressive population able to hamper the immune response against cancer, correlates with tumor progression and overall survival in several cancer types. We have previously shown that MDSCs can be induced *in vitro* from precursors present in the bone marrow and observed that these cells are able to actively proliferate in the presence of activated T cells, whose activation level is critical to drive the suppressive activity of MDSCs. Here we investigated at molecular level the mechanisms involved in the interplay between MDSCs and activated T cells. We found that activated T cells secrete IL-10 following interaction with MDSCs which, in turn, activates STAT3 phosphorylation on MDSCs then leading to B7-H1 expression. We also demonstrated that B7-H1^+^ MDSCs are responsible for immune suppression through a mechanism involving ARG-1 and IDO expression. Finally, we show that the expression of ligands B7-H1 and MHC class II both on *in vitro*-induced MDSCs and on MDSCs in the tumor microenvironment of cancer patients is paralleled by an increased expression of their respective receptors PD-1 and LAG-3 on T cells, two inhibitory molecules associated with T cell dysfunction. These findings highlight key molecules and interactions responsible for the extensive cross-talk between MDSCs and activated T cells that are at the basis of immune suppression.

## INTRODUCTION

Among leukocytes conditioned by tumors, myeloid-derived suppressor cells (MDSCs) represent one of the most important players mediating immunosuppression. MDSCs are immature myeloid cells that fail to complete their differentiation under chronic inflammatory conditions that are typical for the tumor microenvironment. Importantly, these cells acquire strong immunosuppressive functions that allow them to inhibit efficiently T cell mediated anti-tumor reactivity by various mechanisms [1, 2].

Although several hypotheses have been advanced, one of the open issues about human MDSCs is related to the mechanisms driving the expansion in the blood of cancer patients of myeloid suppressive subsets displaying markers and morphology characteristics of either monocytic, granulocytic or immature cells [1]. We demonstrated that G-CSF and GM-CSF allow MDSC expansion from myeloid progenitors present in bone marrow (BM) samples [3]; among these cells, defined as BM-MDSCs, we identified suppressive cells with promyelocitic-like features and named them immature- BM-MDSCs (i-BM-MDSCs). Moreover, we found myeloid cells with the same phenotype in advanced cancer patients and showed that increased circulating levels of these immunosuppressive myeloid cells correlated with worse prognosis and clinically evident disease progression [4]. Over the years, several mechanisms of suppression induced by MDSCs were described both *in vitro* and *in vivo,* indicating that MDSCs exert either direct or indirect immunosuppression of activated T lymphocytes [5]. Among the direct immune suppressive strategies, the most studied is the control of metabolic control of the amino acids L-arginine (L-Arg), L-cysteine, and L-phenylalanine. The two major catabolic enzymes through which MDSCs metabolize L-Arg are arginase (ARG1), which converts L-Arg into urea and L-ornithine, and nitric oxide synthase (NOS), which oxidizes L-Arg generating nitric oxide (NO) and citrulline. ARG1 and NOS are expressed by MDSCs [5] and ARG1 was found up-regulated also in plasma of cancer patients [6]. MDSCs were also shown to act as L-cysteine consumers/sequesters, since these cells import the amino acid but do not express the transporter to release it in the extracellular milieu [7]. Increased NO and up-regulation of reactive oxygen species (ROS) and reactive nitrogen species (RNS) contribute to mediate immune suppression mediated by MDSCs [8]. Furthermore, MDSCs impair T cell viability by expressing ligands of immunoregulatory receptors like PD-L1, both in mice [9-12] and in colorectal cancer patients [13].

STAT3 is a transcription factor implicated in pathways of suppression of different suppressor cells, such as regulatory T cells (Treg), Th17 and also MDSCs [14]. In particular, MDSCs isolated from tumor-bearing mice have increased levels of phosphorylated STAT3, as compared to immature myeloid cells from healthy mice [15], and the expansion of MDSCs is abrogated when STAT3 is inhibited in hematopoietic progenitor cells [16]. Moreover, STAT3 can also induce the expression of S100A8/A9 in murine myeloid cells, which drive further MDSC accumulation and prevent their differentiation [17]. In cancer patients, MDSCs isolated from different anatomical compartments were shown to have high levels of phosphorylated STAT3 that correlated with ARG1 expression, a downstream target of activated STAT3 [18].

We previously observed that i-BM-MDSCs are able to proliferate actively in the presence of activated T cells and that the presence of activated, but not resting lymphocytes, affects MDSC differentiation by blocking their default maturation program, thus rendering them unable to differentiate in mature myeloid cells [4]. In the present study, we further investigated at molecular level the crosstalk between activated T cells and MDSCs and found a loop involving the integrated signals from soluble molecules, transcription factors and surface proteins fuelling the process of immune suppression.

## RESULTS

### T cell-suppression induced by i-BM-MDSCs is the result of bidirectional interactions

We previously demonstrated that some cytokines can drive the generation of an heterogeneous myeloid population, named BM-MDSCs that share not only the phenotype but also the suppressive function of MDSCs isolated from cancer patients. The cell population responsible for immunosuppression is an immature subset resembling to promyelocytes (immature-BM-derived MDSCs, i-BM-MDSCs) while the more differentiated cells (mature-BM-MDSC, m-BM-MDSCs) lack immunosuppressive activity. i-BM-MDSCs are able to proliferate and maintain their immature phenotype only when co-cultured with activated T lymphocytes. We also showed that activated T cells are able to induce changes in MDSC phenotype and sustain their suppressive activity [4].

**Figure 1:**
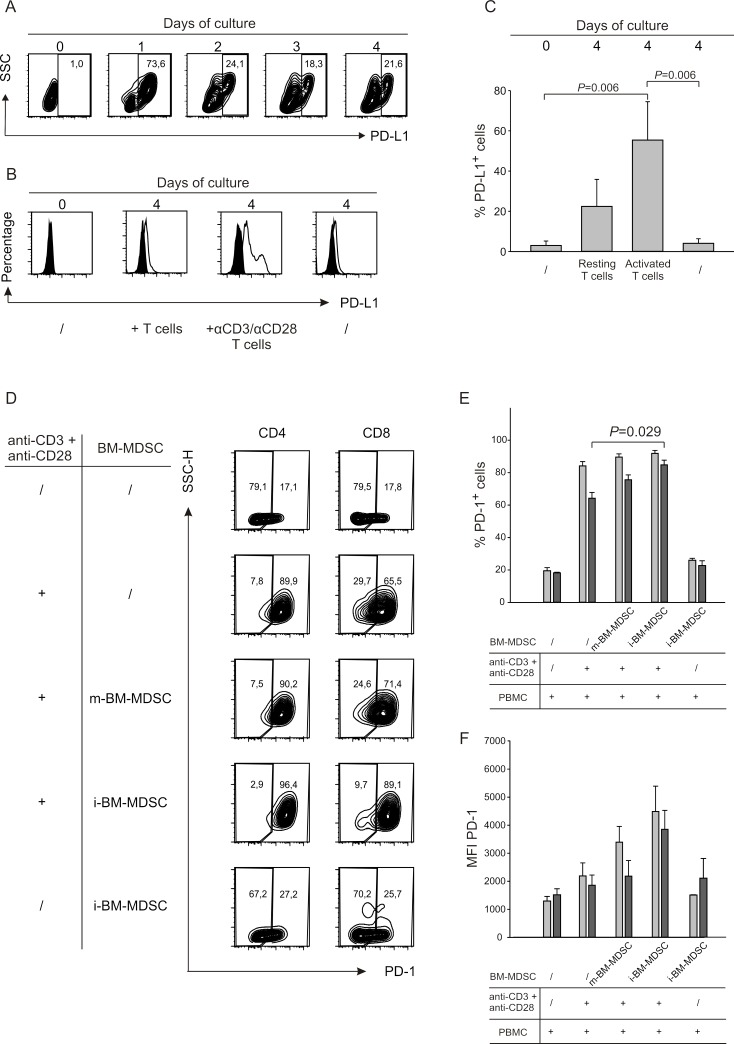

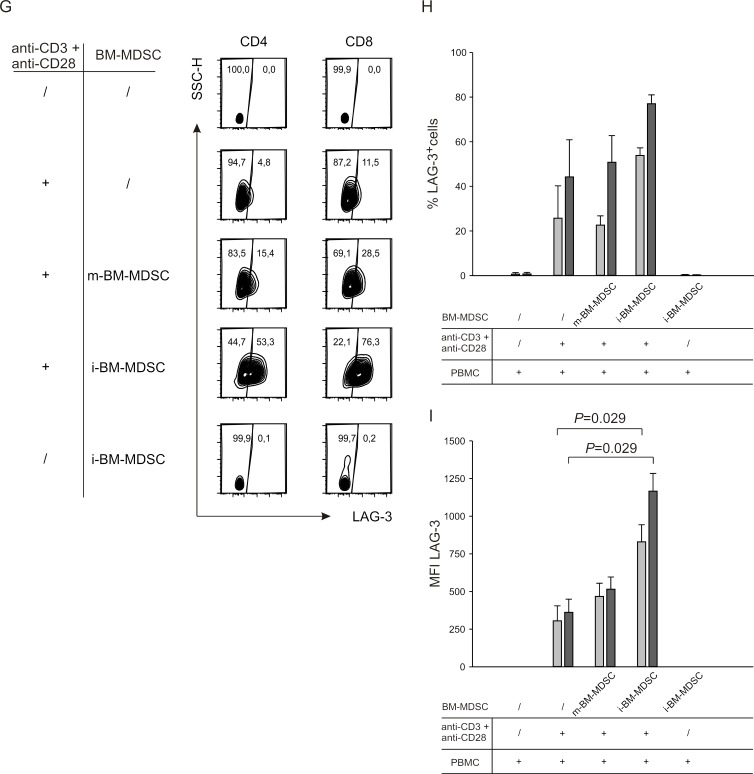
The interaction between activated T cells and i-BM-MDSCs induces expression of surface molecules on both populations **A.** PD-L1 expression was evaluated on i-BM-MDSCs alone (day 0) and at different time points (day 1 to 4) of the co-culture with CellTrace^+^activated T cells. i-BM-MDSCs were gated as CellTrace^−^/CD3^−^. One representative out of four independent experiments is presented. **B.** After four days of co-culture of i-BM-MDSCs with activated T cells, cells were labelled with anti-CD3 and anti-PD-L1 mAbs. Myeloid cells were identified as CellTrace^−^/CD3^−^ cells and the expression of the negative signal (black histogram) was evaluated using a FMO control. The data are representative of 3 independent experiments. **C.** The histograms report the mean percentage of PD-L1^+^ i-BM-MDSCs ± SE of 4 independent experiments. Mann-Whitney U test was applied. **D.** CellTrace^+^ PBMCs were cultured for 4 days alone or in the presence of m-BM-MDSCs and i-BM-MDSCs. Anti-CD3/CD28 antibodies were used to activate T cells. As control, resting T cells were maintained alone or in the presence of i-BM-MDSCs. At the end of the culture, the percentage of PD-1^+^ cells was quantified for CD4 cells gating on CD3^+^/Celltrace^+^/CD8^−^ cells and for CD8 on CD3^+^/CellTrace^+^/CD8^+^ cells. **E.** Percentage of PD-1^+^ cells calculated in CD4^+^ (grey bars) and CD8^+^ (black bars) T lymphocytes. **F.** MFI of PD-1 was calculated on PD-1^+^ cells among CD4^+^ (grey bars) and CD8^+^ (black bars) T cells. Values reported are the mean ± SE of 3 independent experiments. Mann-Whitney U test was applied. **G.** CellTrace^+^ PBMCs were stimulated with anti-CD3/CD28 in the presence of m-BM-MDSCs and i-BM-MDSCs. At the end of the culture, LAG-3 expression was analyzed in the CD3ε^+^/CellTrace^+^/CD8^−^ gate (CD4) and in the CD3ε^+^/CellTrace^+^/CD8^+^ gate (CD8). The panel represents the frequency of LAG-3^+^/CD4^+^ and of LAG-3^+^/CD8^+^ cell subsets on resting or stimulated T cells co-cultured with m-BM-MDSCs and i-BM-MDSCs. The figure shows a representative experiment out of 3 performed. **H.** Percentage of LAG-3^+^ cells among CD4^+^ (grey bars) and CD8^+^ (black bars) subsets. **I.** LAG-3 MFI calculated on CD4^+^ (grey bars) and CD8^+^ (black bars) cell subsets. Data represent mean ± SE of 3 independent experiments. *P* < 0.001, Mann-Whitney U test.

To unveil the molecules involved in immunoregulatory pathways, we monitored the expression of B7 family members in i-BM-MDSCs following contact with activated T cells. Interestingly PD-L1 (also named B7-H1) and B7-H3, but not B7-H2, were significantly upregulated only after cell to cell contact with stimulated T cells (data not shown). Since the ligand of B7-H3 is not known yet, we focused on PD-L1 and evaluated the kinetics of its expression on MDSCs over 4 days of culture with activated T cells. By flow cytometry, we observed a strong induction of PD-L1 on the first day of cell culture, which then decreased and was maintained until the fourth day (Figure 1A). Of note, only the activated T cells were able to increase significantly PD-L1 expression on myeloid cells, since a negligible effect was observed with resting T cells (Figure 1B). Cumulative data shown in Figure 1C confirm a significant increase in the percentages of PD-L1^+^ cells among MDSCs when in contact with activated T cells.

Since the receptor of PD-L1 is PD-1, we analysed the expression of this protein on both CD4^+^ and CD8^+^ T cells under the same conditions. As expected, PD-1 was expressed at low levels in resting T cells while T cell activation caused a robust increase in its expression, which was not affected by the addition in culture of m-BM-MDSCs (Figure 1D and 1E). Instead, the presence of i-BM-MDSCs caused a significant increase not only in the percentage of PD-1^+^ cells, but also in the intensity of PD-1 expression on CD8^+^ but not CD4^+^ T cells (Figure 1E and 1F). These results show that PD-1 expression is mainly driven by T cell activation, although contact with suppressive MDSCs can further up-regulate this process on CD8^+^ T cells.

Another marker upregulated on i-BM-MDSCs following contact with activated T cells is HLA-DR [4]. We speculated that this marker might also be involved in i-BM-MDSC-mediated immune suppression and indeed, beside the antigen presentation, additional functions have been attributed to MHC class II molecules. Interestingly, the negative co-stimulatory receptor LAG-3 is a natural ligand for MHC class II [19, 20], and recent preclinical studies documented a role for LAG-3 in CD8^+^ T cell exhaustion [21-23].

We thus evaluated LAG-3 expression on CD4^+^ and CD8^+^ cells after contact with MDSC cell subsets, and found a strong up-regulation of this marker especially on CD8^+^ T cells (Figure 1G). Expression of LAG-3 is induced specifically by i-BM-MDSCs, as evaluated by percentage and by mean fluorescence intensity (MFI), since cell culture with non-suppressive subset of m-BM-MDSCs does not lead to a significant increase of expression of LAG-3 (Figure 1H and 1I). Overall, these results indicate that there is a dynamic interplay between MDSCs and activated T cells, as some surface receptors and their respective ligands are modulated on both cell types when these two cells come into contact.

### Characteristics of T cell dysfunctions induced by MDSCs

**Figure 2:**
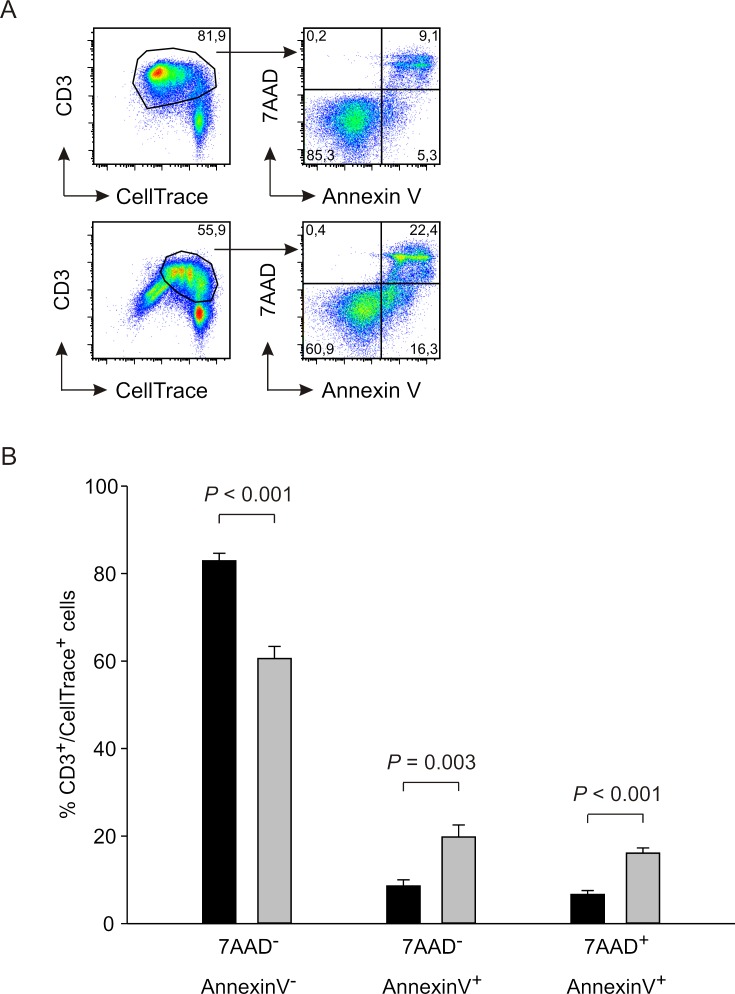
Analysis of apoptosis induction by i-BM-MDSCs on activated T cells **A.** CellTrace-labelled PBMCs were cultured alone (upper panels) or in the presence of i-BM-MDSCs for 4 days (lower panels). T lymphocytes were gated as CellTrace^+^/CD3^+^ cells and then apoptotic (7AAD^+^/ Annexin V^+^), early apoptotic (7AAD^−^/Annexin V^+^) and live (7AAD^−^/Annexin V^−^) cells were analyzed. **B.** Mean ± SE of 8 independent experiments. Black bars refer to activated T cells alone, grey bars to activated T cells in the presence of i-BM-MDSCs.

It is well known that PD-1 and LAG-3 are inhibitory receptors involved in the phenomenon of T cell exhaustion, whose main feature is the loss of function of CD8^+^ and CD4^+^ T cells [24]. To gain insight in the dysfunction induced by MDSCs on T cells, we determined the extent of T cell apoptosis induced by i-BM-MDSCs. To this aim, we cultured anti-CD3/CD28 activated T cells with i-BM-MDSCs and evaluated apoptosis after 4 days by 7AAD and Annexin V staining, followed by flow cytometry analysis thus discriminating between apoptotic (7AAD^+^/Annexin V^+^), early apoptotic (7AAD^−^/Annexin V^+^) and live (7AAD^−^/Annexin V^−^) cells (Figure 2A). As expected, T cell proliferation was significantly reduced in the presence of MDSCs, and accordingly we observed a significant increase in apoptotic and early apoptotic T cells (Figure 2B, grey bars), as compared to T cells cultured without myeloid cells (Figure 2B, black bars). However, the majority of the cells in culture were alive, expressing the markers of exhaustion PD-1 and LAG-3 (Figure 1) and restrained in their proliferative capacity (Figure 2A). Thus, these results, suggest that induction of apoptosis is not the main mechanism by which MDSCs exert immunosuppression.

### Role of IL-10 in MDSC-induced T cell suppression

**Figure 3:**
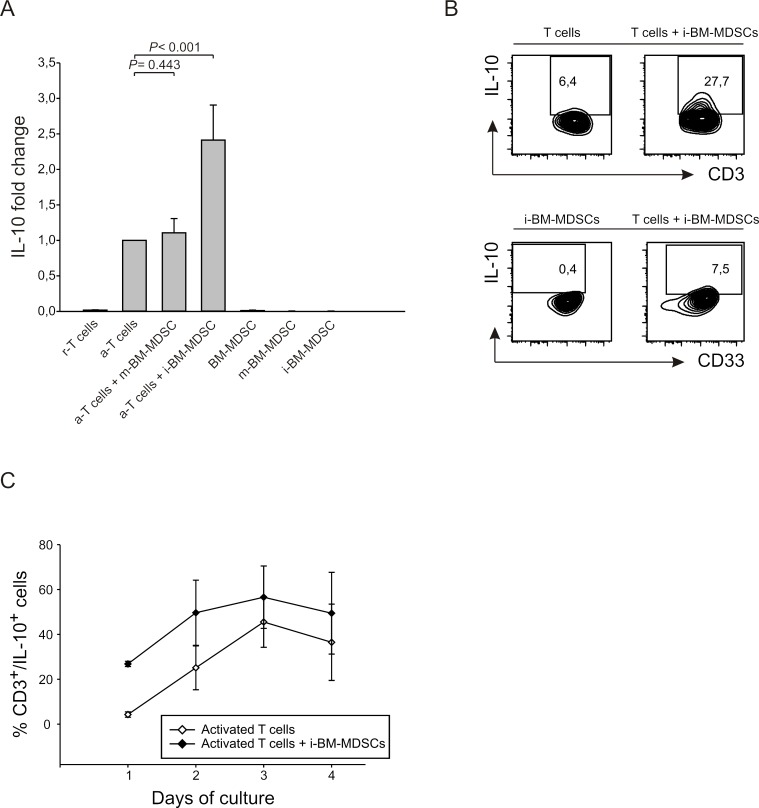
IL-10 is released from suppressed T cells **A.** CellTrace-labelled PBMCs were activated alone or in the presence of m-BM-MDSC and i-BM-MDSC cell subsets for 4 days, added at a ratio of 1:1. ELISA assay was performed on the supernatants collected. r-T cells = unstimulated T cells, a-T cells = anti-CD3/CD28 T cells. In each experiment, IL-10 concentration was normalized on anti-CD3/CD28 T cells and expressed as fold increase. The histogram shows the mean ± standard error (SE) of 10 independent experiments. Mann-Whitney U test was applied. **B.** IL-10 secretion assay was performed on i-BM-MDSCs cultured alone (left lower panel) and on CellTrace^+^ PBMCs stimulated with anti-CD3 and anti-CD28 for one day, alone (left upper panel) or in the presence of i-BM-MDSCs (right panels). Anti-CD3 and anti-CD33 mAbs allowed to discriminate between CD3^+^/CellTrace^+^ T cells (upper panels) and CD33^+^/CellTrace^−^ myeloid cells (lower panels). To determine the percentage of IL-10^+^ cells, gating was set on FMO control. **C.** Kinetics of IL-10 secretion in cell cultures of activated T cells with (black dots) or without (white dots) i-BM-MDSCs. The figure shows a representative experiment out of 3 performed.

We next focused our attention on soluble factors involved in immune suppression, and in particular on IL-10, a cytokine with inhibitory effects on the immune system. We thus evaluated IL-10 production during the co-culture of activated T cells with MDSCs. We cultured CellTrace-labelled PBMCs activated with anti-CD3/CD28 for four days in the presence or absence of myeloid cells and collected supernatant for IL-10 detection. IL-10 was released by activated T cells and significantly increased in the presence of suppressive i-BM-MDSCs, whereas m-BM-MDSCs did not alter IL-10 production (Figure 3A). Since this assay does not discriminate whether myeloid or lymphoid cells are responsible for IL-10 secretion, we performed an IL-10 secretion assay that discriminated IL-10^+^ cells among either T lymphocytes or myeloid cells, by gating respectively on CD3^+^ and on CD33^+^ cells. Results indicate that activated T cells are the cell population mainly responsible for IL-10 secretion under these conditions, while the contribution of myeloid cells is very low (Figure 3B). Moreover, the percentage of CD3^+^/IL-10^+^ cells is increased when activated T cells are cultured in the presence of MDSCs, suggesting that the presence of suppressive cells upregulates IL-10 expression (Figure 3C). As far as the kinetics of IL-10 release was concerned, we observed that the cytokine is rapidly released after one day of culture and its secretion increases up to the third day, then reaching a plateau (Figure 3C). At each time point, IL-10 release by T cells in the presence of MDSCs was always higher. Taken together, these results indicate that the presence of MDSCs induces IL-10 production by activated T cells and that IL-10 secretion is an early event that occurs during the interaction between the two populations.

### STAT-3 activation and PD-L1 expression on MDSCs following interaction with activated T cells

**Figure 4:**
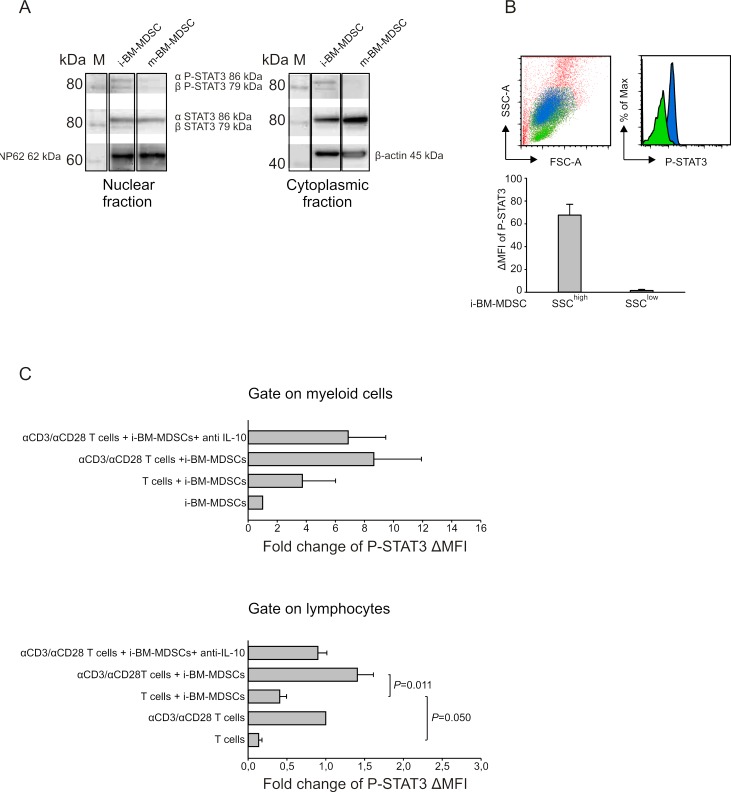
Analysis of STAT3 phosphorilation on i-BM-MDSCs **A.** Western blot analysis of P-STAT3 and STAT3 isoforms' expression in the nuclear (left panel) and cytoplasmic (right panel) protein fractions of i-BM-MDSCs and m-BM-MDSCs. Nucleoporin 62 (Np62) and β-actin were used as endogenous controls respectively for nuclear and cytoplasmic fractions. Molecular weight of the proteins was determined on the basis of a chemiluminescent marker (M). **B.** Intracellular staining for P-STAT3 was performed on i-BM-MDSCs and the MFI was evaluated by subtracting the signal of secondary antibody alone (ΔMFI). The morphology of SSC^high^(blue) and SSC^low^(green) cells and the relative P-STAT3 expression is shown in the upper panels. These results are representative of 4 independent experiments. **C.** i-BM-MDSCs were cultured for 20 hours in the presence of resting or anti-CD3/anti-CD28 activated T cells. To discriminate between myeloid cells and T cells in co-culture, cells were gated respectively on CellTrace^−^ and CellTrace^+^ cells and the ΔMFI of P-STAT3 was evaluated as described above. Values obtained were then normalized on the ΔMFI of i-BM-MDSCs cultured alone for 20 hours, when considering myeloid cells (upper panel), and on the ΔMFI of activated T cells, when evaluating T lymphocytes (lower panel). Anti-IL-10 mAb (10 μg/ml) was added to the co-culture between activated T cells and i-BM-MDSCs. The values reported are presented as mean ± standard error (SE) of 3 independent experiments. Student's t test was applied.

Subsequently, we explored the mechanisms set in motion by IL-10. We focused our attention on STAT3, a transcription factor activated by IL-10 [25] and involved in immunosuppression. To this aim, we performed a Western Blot analysis of the nuclear and cytoplasmic fractions of proteins extracted both from m-BM-MDSCs and i-BM-MDSCs and evaluated phosphorylation at Tyr705 on both α and β isoforms (Figure 4A). Data indicate that, despite a similar presence of STAT3 in both suppressive and not suppressive myeloid cells, STAT3 phosphorylation was detected only in the nuclear fraction of suppressive i-BM-MDSCs, thus demonstrating STAT3 activation in this cell subset (Figure 4A). We also evaluated by flow cytometry P-STAT3 expression in i-BM-MDSCs and observed that two cell populations can be distinguished on the basis of their different morphology, one showing a high side-scatter (SSC), in blue, and the other with a lower SSC (Figure 4B), in green. Interestingly these two cell populations differed in terms of STAT3 phosphorylation, being SSC^high^ cells the only positive for P-STAT3 (Figure 4B).

We next investigated by flow cytometry whether STAT3 activation is influenced by the presence of T cells. To this aim, we evaluated P-STAT3 MFI in the SSC^high^ subset of i-BM-MDSCs, 20 hours after the contact with either resting or activated T cells (Figure 4C, upper panel). This analysis revealed a trend in the increase of P-STAT3 expression in myeloid cells in the presence of anti-CD3/CD28 activated T cells, in comparison to the presence of resting T cells (Figure 4C, upper panel). We then explored whether the interaction with MDSCs also modulates STAT3 activation in T cells. Indeed, we found a significant increase in STAT3 phosphorylation within T cells stimulated in the presence of i-BM-MDSCs (Figure 4C, lower panel). These results suggest the existence of an interplay between activated T cells and MDSCs that leads to the activation of STAT3 signaling pathway in both cell partners. To prove the relationship between STAT3 activation and IL-10 release, we added a neutralizing mAb against IL-10 and noticed a decrease of STAT3 phosphorylation on both myeloid (Figure 4C, upper panel) and activated T cells (Figure 4C, lower panel), thus confirming this connection.

**Figure 5:**
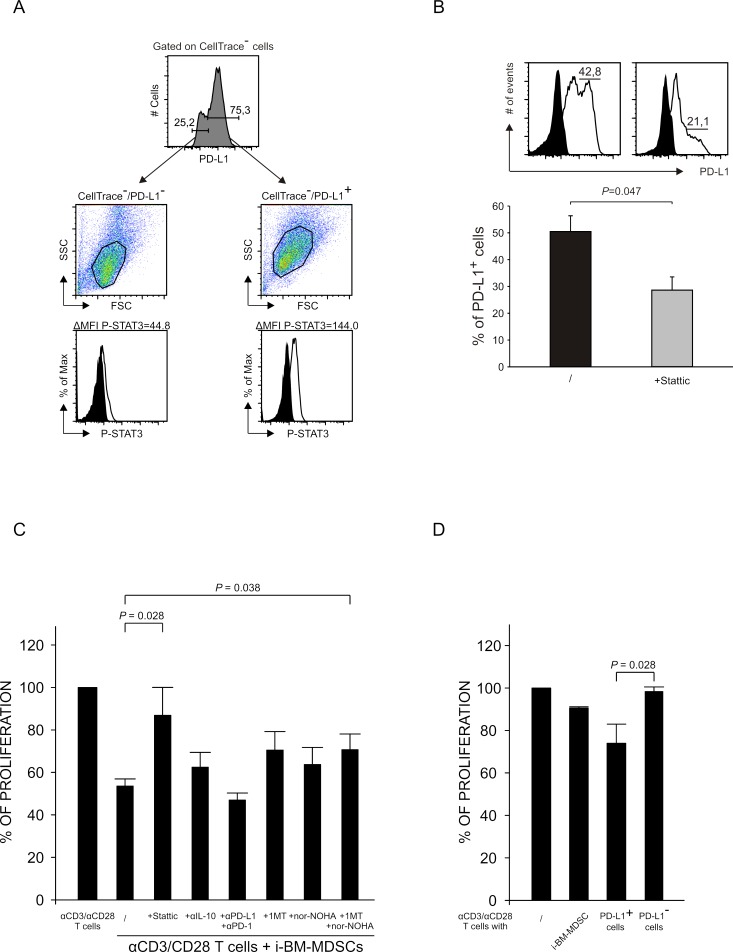
Relationship between STAT3 activation and PD-L1 expression **A.** After 20 hours of co-culture of i-BM-MDSCs and T cells, PD-L1^+^ and PD-L1^−^ myeloid cells were separated by FACS sorting and an intracellular staining for P-STAT3 was performed. Black histograms show the signal of secondary antibody alone. The results are representative of 3 independent experiments. **B.** i-BM-MDSCs untreated (black histogram) or pretreated (grey histogram) with 5 μM Stattic for 30 minutes were cultured for 20 hours with CellTrace^+^ activated T cells. The percentage of PD-L1^+^ cells among CellTrace^−^ myeloid cells was quantified as shown in the upper panel (black histograms are the FMO control). Lower graph represent mean ± SE of 3 independent experiments. Student's t test was applied. **C.** CellTrace^+^ PBMCs were cultured for 4 days alone and in the presence of i-BM-MDSCs, pretreated for 30 minutes with 5 μM Stattic, while mAbs anti-IL-10 (10 μg/ml), anti-PD-1 and anti-PD-L1 (1 μg/ml), nor-NOHA (0.5 mM) and 1-MT (1 mM) were added at the beginning of the culture. After 4 days, T cells were stained with anti-CD3 antibody. Immunosuppressive activity of i-BM-MDSCs was evaluated considering the percentage of proliferating CD3^+^ T cells. For each experiment, the values were normalized on the proliferation of activated T cells alone. Mean ± SE is reported. Student's t test was applied. **D.** After 20 hours of co-culture of i-BM-MDSCs with activated T cells, PD-L1^+^ and PD-L1^−^ subsets and total myeloid cells were separated by FACS sorting and cultured with pre-activated T cells for 3 days. Immunosuppressive activity was calculated as described for 3C. The histograms report the mean of 3 independent experiments ± SE. Student's t test was applied.

It is known that STAT3 can induce the expression of PD-L1 by binding to its promoter and leading to the transcription of the gene [26]. Besides, also IDO1 can induce the expression of PD-L1 and, in fact, we found that IDO1 expression is highly increased after contact with activated T cells (Supplementary Figure 1). To evaluate the link between P-STAT3 and PD-L1, we investigated whether these two markers were co-expressed in the same cells. To this aim, we cultured for 20 hours MDSCs with activated T cells and then sorted PD-L1^+^ and PD-L1^−^ cells by FACS. Next, we analyzed STAT3 phosphorylation by intracellular staining on sorted cells. As shown in Figure 5A, PD-L1^+^ cells had a higher intensity of P-STAT3 expression, as compared to PD-L1^−^.

Based on this result, we investigated whether the inhibition of STAT3 phosphorylation and therefore its activation had any effect on the expression of PD-L1. To this aim, we evaluated the percentage of PD-L1^+^ MDSCs pretreated with Stattic, an inhibitor of STAT3 phosphorylation at Tyr705, after contact with activated T cells. We observed that the PD-L1-expressing cells were significantly reduced in the presence of Stattic, thus supporting the concept that STAT3 activation induces PD-L1 expression (Figure 5B).

### The immunosuppression mediated by i-BM-MDSCs depends on STAT3 activation and the expression of IDO and arginase

We demonstrated that IL-10, STAT3 and PD-L1 are interrelated in a loop that is active in immunosuppressive cells after contact with activated T cells. We therefore asked whether inhibitors of IL-10, STAT3 and PD-L1 were able to rescue the proliferation of T cells suppressed by MDSCs. To this aim, we used IL-10 blocking mAbs, and the combination of anti-PD-L1 and anti-PD-1 mAbs to block in full the interaction of PD-L1, expressed by myeloid cells, with its receptor PD-1 on activated T cells. Instead, we could pretreat only i-BM-MDSCs with Stattic before adding them to the co-cultures, since Stattic was toxic on T cells even at very low concentrations. Moreover, since PD-L1 is known to be induced by IDO whose expression is highly increased after interaction with activated T cells (Supplementary Figure 1), we also evaluated the effect of IDO inhibitor 1-Methyl-Tryptophan (1-MT). Lastly, we tested the arginase inhibitor nor-NOHA, since i-BM-MDSCs also express the immunosuppressive enzyme ARG1 [4]. Our results demonstrate that the pretreatment with Stattic or the addition of the combination of both nor-NOHA and 1-MT were able to rescue the proliferation of T cells (Figure 5C) thus indicating that STAT-3, ARG1 and IDO play a relevant role among the mechanisms that underlie i-BM-MDSC-mediated immunosuppression. Surprisingly, we found no significant recovery of T cell proliferation when either anti-IL-10 or anti-PD-1/anti-PD-L1 mAbs were added to the cell cultures.

When we further addressed the relevance of PD-L1 expression in MDSC function, we observed that only sorted PD-L1^+^ i-BM-MDSCs were responsible for the immune suppression (Figure 5D), while PD-L1^−^ cells were not able to interfere with T cell proliferation. In this case, the combination of nor-NOHA and 1-MT restored T cell proliferation (data not shown), thus confirming the role of arginase and IDO in MDSC-mediated T cell suppression.

### Functional and phenotypical analysis of myeloid and T cells infiltrating tumor metastases

**Figure 6:**
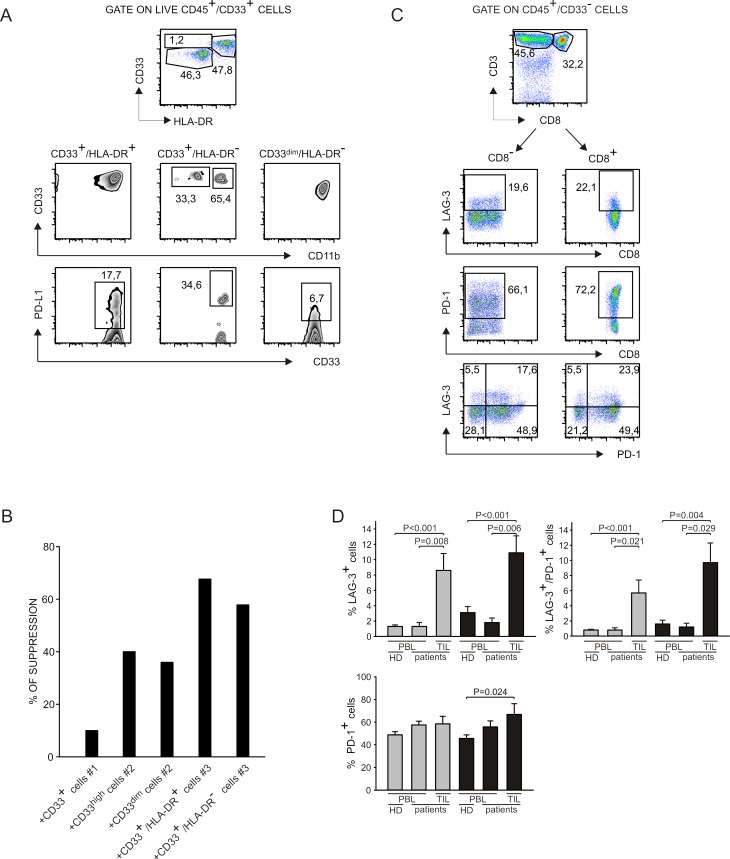
Analysis of myeloid cells and T lymphocytes at tumor site **A.** Cell suspensions were obtained from liver metastases of colorectal cancer patients and from draining lymph nodes of melanoma patients and stained with livedead, anti-CD45, anti-CD33, anti-HLA-DR, anti-CD11b and anti-PD-L1. Live myeloid cells were gated as livedead^−^/CD45^+^/ CD33^+^ and then three populations were analyzed on the basis of HLA-DR expression. In each population, the expression of CD11b and of PD-L1 was evaluated. Data represent one out of 5 independent experiments. **B.** Cell suspensions obtained from biopsies were stained with anti-CD45, anti-CD33 and anti-HLA-DR. Myeloid cells were gated as CD45^+^/CD33^+^ and sorted by FACS. For patients #2 and #3 we were able to separate distinct subpopulations that are, respectively, CD33^high^ and CD33^dim^; CD33^+^/HLA-DR^+^ and CD33^+^/HLA-DR^−^. Cells were cultured for 4 days in the presence of CellTrace^+^ activated PBMCs. Immunosuppression of these populations was evaluated on activated T cells, (gated as CellTrace^+^/CD3^+^), by considering the absolute number of T cells, normalized on the control without myeloid cells. **C.** Cell suspensions were obtained from liver metastases of colorectal cancer patients and from draining lymph nodes of melanoma patients and stained with livedead, anti-CD45, anti-CD33, anti-CD3, anti-CD8, anti-PD-1 and anti-LAG-3. Lymphocytes were gated as livedead^−^/CD45^+^/CD33^−^ cells and then the expression of LAG-3 and PD-1 was evaluated in CD3^+^/CD8^−^ and CD3^+^/CD8^+^ subsets. **D.** Peripheral blood of healthy donors and of melanoma and colorectal cancer patients was stained with anti-CD3, anti-CD8, anti-PD-1 and anti-LAG-3 and analyzed by gating on CD3^+^/CD8^+^ and on CD3^+^/CD8^−^ populations to detect LAG-3 and PD-1 expression. The histograms report the mean and SE of the percentage of LAG3^+^, LAG-3^+^/PD-1^+^ and PD-1^+^ cells calculated among CD8^−^ (grey bars) and CD8^+^ (black bars) T cells in the peripheral blood of healthy donors (*n* = 23) and of melanoma and colorectal cancer patients (*n* = 6) and at the tumor site of the same patients (*n* = 9). Mann-Whitney U test was applied.

We studied myeloid and lymphoid cells present in liver metastases of colorectal cancer patients and in metastatic lymph nodes of melanoma patients to analyze the immune suppressive contexture *in vivo*. We set up a multicolor flow cytometry staining by which we were able to analyze three different myeloid populations that differed for HLA-DR expression, and that are consistent with the phenotype of monocytic (CD33^+^/HLA-DR^+^/CD11b^+^) and granulocytic (CD33^+^/HLA-DR^−^/CD11b^dim/+^ and CD33^dim^/HLA-DR^−^) MDSCs (Figure 6A, upper panels). Interestingly, we observed that PD-L1 was expressed by all the myeloid subsets, although at different levels (Figure 6A, lower panels) and that CD33^+^/HLA-DR^−^ cells presented a down-regulation of CD11b similar to i-BM-MDSCs [4]. To test the immunosuppressive activity of myeloid cells infiltrating tumors, we sorted CD33^+^ cells and cultured them for four days with allogeneic activated T cells.

As shown in Figure 6B, four myeloid cell subsets isolated from two different patients were able to affect T cell proliferation. For two patients, we sorted distinct CD33^+^ cell subsets on the basis of CD33 intensity (patient #2) or of HLA-DR expression (patient #3), but in every case all the sorted cells showed the same suppressive activity, while CD33^+^ cells from an additional patient (patient #1) exerted lower immunosuppressive activity.

Since the markers PD-1 and LAG-3 were up-regulated on T cells *in vitro* following contact with i-BM-MDSCs, we tested whether these molecules were expressed also by TILs. Following the gating strategy showed in Figure 6C, we observed that PD-1 levels were very high in all the samples tested and that also LAG-3 is expressed in most of the patients analyzed, although to a lower extent, both on CD4^+^ and on CD8^+^ T cells (Figure 6C and 6D). Levels of LAG-3 and PD-1 were significantly higher on T cells present in the tumor microenvironment, compared to circulating cells of the same patients (Figure 6D). Collectively, these data indicate that myeloid suppressive cells consistent with the phenotype of MDSCs are present in tumor metastases, along with TILs expressing PD-1 and LAG-3 markers.

## DISCUSSION

It is becoming increasingly apparent that generation and functional activity of MDSCs are two separate events, driven by different types of signals. We previously analyzed the factors driving MDSC expansion from bone marrow precursors and found that normal promyelocytes, present in the bone marrow, can be primed with tumor-derived factors to acquire a suppressive phenotype and become MDSCs and C/EBPβ is a key transcription factor controlling this process [3]. In the present study, we explored the mechanisms driving MDSC-mediated immune suppression in i-BM-MDSCs. We observed that MDSCs are harmless when in contact with resting T cells and become functionally active only in the presence of activated T cells, a crucial interaction able to induce a number of events ranging from IL-10 release, STAT3 activation, PD1, PD-L1 and LAG-3 up-regulation.

We found that IL-10 is released by activated T cells and this, in turn, leads to the activation of STAT3 in both cell types. Interestingly, a similar mechanism was reported also for mouse macrophages, stimulated with CpG, interacting with CD4^+^ T cells activated by anti-CD3/CD28. Under these conditions, the cell-to-cell contact led to an increase in IL-10 production in both cell types and a STAT3 inhibitor abrogated CD4^+^ T cell production of IL-10 [27].

We demonstrated that STAT3 activation was sustained by IL-10 signalling, although a low level of activation was observed also in MDSCs before the contact with activated T cells. This was probably dependent on the fact that MDSCs were derived by using the combination of G-CSF and GM-CSF, cytokines known to signal through STAT3 [28, 29]. Studies performed in a mouse model with conditional deletion of STAT3 in the bone marrow proved that hematopoietic progenitor cells and myeloid precursors deleted for STAT3 were refractory to G-CSF effects. These studies also demonstrated that STAT3 directly controls G-CSF-dependent expression of C/EBPβ [30], a transcription factor with a crucial role in both emergency granulopoiesis and MDSC immunosuppressive activity [3]. Furthermore, conditional STAT3 gene disruption in myeloid cells brought about a significant reduction in granulocytic MDSCs [31]. As far as the mechanism of action is concerned, STAT3 can modulate MDSC expansion by the up-regulation of the protein S100A9, a member of the large family of S100 proteins that forms a dimer with S100A8. The up-regulation of this protein in hematopoietic progenitor cells of colon carcinoma-bearing mice was associated with the expansion of MDSCs and impaired differentiation of DCs [17]. CD14^+/^S100A9^+^ MDSCs expanded in NSCLC patients and suppressed T cell proliferation via iNOS, ARG1, IL-10 and the IL-13/IL-4Rα pathway [32]. ARG1 was expressed also on CD14^+^/HLA-DR^low/−^ MDSCs in HNSCC patients and STAT3 activation led to ARG1 expression by binding to its promoter [18]. Therefore, STAT3 can regulate MDSC expansion and activity by activating different signalling pathways.

In this study, we also demonstrated that STAT3 phosphorylation leads to the expression of PD-L1, a molecule that can negatively regulate immune responses by interacting with its receptor PD-1 on lymphocytes. PD-L1 was expressed on MDSCs obtained from ascites and spleens of mice bearing an ovarian carcinoma and caused immunosuppression by interacting with PD-1 on Treg cells [12]. In *ret* oncogene-dependent, autochthonous model of melanoma, the expression of PD-L1, B7-H3 and B7-H4 on MDSCs was dependent on the interaction between MDSCs and Treg cells [11]. We focused our attention on PD-L1 since STAT3 binding sites are present on the promoter of PD-L1 gene [26]. Moreover, studies performed on patients infected with HIV revealed that, during infection, PD-1 is up-regulated on monocytes and the interaction of PD-1 with PD-L1, expressed on other cell types, induced IL-10 production, which in turn led to CD4^+^ T cell dysfunction [33]. Moreover, IL-10 can modulate PD-L1 up-regulation on human macrophages during HIV infection [34]. To the best of our knowledge, this is the first indication that a signalling pathway driven by IL-10, through the activation of STAT3, leads to the expression of PD-L1 in human MDSCs. A similar loop was described for human monocyte-derived DCs differentiated in the presence of TLR agonists. These DCs acquired a tolerogenic function that was dependent on MAPK-induced IL-6 and IL-10 production, which drives STAT-3 mediated PD-L1 expression [35]. We speculate that also in MDSCs there could be a possible involvement of IL-6 signalling, since we have preliminary evidence that this cytokine is induced in MDSCs following interaction with activated T cells (unpublished data).

A recent study reported that MDSCs present in breast cancer tissues inhibited T cell proliferation and induced apoptosis in T cells in an IDO-dependent manner. IDO expression was up-regulated in MDSCs induced from healthy donor umbilical cord blood [36] and its expression was dependent on STAT3 activation [37]. In line with these results, our data also suggest IDO involvement in MDSC inhibitory activity, since IDO expression is significantly increased in i-BM-MDSC and its inhibition, in combination with ARG1 inhibitor, results in a significant recovery in T cell proliferation. Since immune suppression depends on the expression of ARG1 and IDO, these results open the possibility to combine specific inhibitors of these enzymes for the therapy of cancer.

Besides the characterization of the molecular mechanisms active in MDSCs, in this study we also evaluated the fate of suppressed T cells. PD-1 and LAG-3, two markers associated with T cell exhaustion were indeed upregulated in T cells cultured with MDSCs, with some differences: PD-1 on suppressed CD8^+^ T cells and LAG-3 on both CD4^+^ and CD8^+^ T cells. LAG-3 expression is negatively correlated with T cell proliferation, activation and homeostasis, and it is known to interact with high affinity with MHC class II. Blocking LAG-3/MHC class II interaction by anti-LAG-3 mAbs increased the number of CD4^+^ and CD8^+^ T cells entering division, after stimulation with APCs and low antigen concentrations [38]. Interestingly, i-BM-MDSCs increase the expression of MHC class II following contact with activated T cells [4], and thus an interaction between HLA class II and LAG-3 could be a critical mechanism mediating immunosuppression. This hypothesis is supported by mouse models demonstrating that CD4^+^ T cell tolerance depended on MHC class II expression. Interestingly, cell-to-cell contact between CD4^+^ T cells and MDSCs could enhance the immunosuppressive activity of MDSCs by cross-linking of MHC class II [39].

The negative effect of MHC class II/LAG-3 interaction on T cell functionality might be enhanced by the binding of PD-L1, expressed on MDSCs, to its receptor PD-1, present on activated T lymphocytes. The synergistic effect of PD-1 and LAG-3 in promoting tumor immune escape is suggested by studies on CD8^+^ T cells specific for NY-ESO-1 present in peripheral blood and tumor site of patients with ovarian cancer. Interestingly, tumor-derived, NY-ESO-1-specific CD8^+^ T cells had an impaired effector function and enriched co-expression of LAG-3 and PD-1, as compared to peripheral blood CD8^+^ T lymphocytes. Expression of LAG-3 and PD-1 was up-regulated by IL-10, IL-6 and tumor-derived APCs [40]. Moreover, treatment with anti-LAG-3/anti-PD-1 combination immunotherapy demonstrated higher efficacy in tumor eradication and the enhancement of adaptive immune responses as compared to monotherapy [41]. Thus, regulation of pathways involved in T cell functional impairment mediated by MDSCs could be deeply connected to IL-10 signalling. Consistent with these results, we found high levels of PD-1^+^ and of LAG3^+^ T cells in the microenvironment of advanced tumors, where they can engage inhibitory signals from myeloid cells expressing PD-L1 and MHC class II. Moreover the presence within the tumors of myeloid cells with the phenotype and functional activity of MDSCs, supports the hypothesis that the PD-1/PD-L1 and LAG-3/HLA class II interactions might be pivotal to the immune deviation in the tumor microenvironment.

## MATERIALS AND METHODS

### BM-MDSC generation and cell subsets sorting

Fresh Bone Marrow (BM) aspirates with normal cytological characteristics were obtained from patients enrolled in the protocol AIEOP-BFM-ALL 2000 [4]. BM samples were also obtained from patients undergoing surgical implants in the Orthopaedic Clinic. Both studies were approved by Ethics Committee and patients gave their informed consent.

BM aspirates were subjected to lysis to remove red blood cells, with a hypotonic solution of ammonium chloride. Myeloid fractions were separated through cell sorting and a cell pellet was frozen to isolate RNA to perform reverse transcription (RT) and real-time RT-PCR. Briefly, single cell suspensions of *ex-vivo* BM or BM-MDSCs were stained with anti-CD11b PE (Beckman Coulter, CA, USA), anti-CD16 FITC (Beckman Coulter) and anti-CD3ε PC7 (Beckman Coulter,) and sorted on a FACSAria (Becton Dickinson, NJ, USA). CD11b^low/−^/CD16^−^ cells were isolated excluding lymphocytes on the basis of CD3 expression and forward/side scatter parameters. CD11b^low/−^/CD16^−^ BM-MDSCs were separated also after 1 day of co-culture with activated T cells (previously stained with CellTrace) by labelling cells with anti-CD3ε PC7 and sorting myeloid cells contained in the gate CD3^−^/CellTrace^−^. All the fractions were obtained with a purity of at least 95%.

For functional assays, *ex-vivo* BM samples were depleted of CD3^+^/CD19^+^/CD56^+^ lymphocytes by immunomagnetic beads (Miltenyi Biotec, Bergisch Gladbach, Germany) and plated at 2×10^6^ cells/well into a 24-well tissue culture plate (Becton Dickinson, NJ, US) in IMDM (Iscove's Modified Dulbecco's Medium, Gibco Invitrogen, California, USA) supplemented with 10% FBS (Fetal Bovine Serum, Gibco), 0,01 M HEPES, 0,55 mM Arginine (Sigma-Aldrich), 0,24 mM Asparagine (Sigma-Aldrich) and 1,5 mM Glutamine (Sigma-Aldrich), penicillin/streptomycin and β-mercaptoethanol. Cells were cultured with 40 ng/ml of both G-CSF and GM-CSF for four days at 37°C, 8% CO^2^, in order to obtain BM-MDSCs, as previously described [4]. Recombinant GM-CSF and G-CSF were purchased from Miltenyi Biotec (Bergisch Gladbach, Germany). After four days, BM-MDSCs were depleted of CD11b^+^ cells with immuno-magnetic anti-human CD11b beads (Miltenyi Biotec), in order to isolate CD11b^low/−^/CD16^−^ cells (i-BM-MDSCs) and CD11b^+^/CD16^+^ (m-BM-MDSCs) cells. The purity of sorted cells was checked by staining both fractions with anti-CD16 FITC (BD Pharmingen) and anti-CD11b PE (Beckman Coulter) antibodies and analyzing cells by FACSCalibur cytometer (BD Biosciences).

### CellTrace labelling and proliferation assay

Peripheral blood mononuclear cells (PBMCs) were isolated from the peripheral blood of healthy donors by density gradient centrifugation on Ficoll-Paque PLUS (GE Healthcare-Amersham, NJ, USA), as previously described [42]. PBMCs were stained with 0.5 μM CellTrace™ Violet Cell Proliferation Kit (Invitrogen, Molecular Probes, MA, USA), according to manufacturer's instructions. CellTrace-labelled PBMCs were activated with coated 1 μg/ml anti-CD3 and 5 μg/ml soluble anti-CD28 (BioLegend, CA, USA) for four days and co-cultured in flat bottom 96 well plates at the 1:1 ratio with cell subsets sorted from BM-MDSCs. Cell cultures were incubated at 37°C and 5% CO_2_ in arginine-free-RPMI (Biological Industries, Kibbutz Beit Haemek, Israel), supplemented with 150 μM arginine, 10% FBS, 10 U/ml penicillin and streptomycin, and HEPES. In selected experiments, anti-IL-10 (BioLegend) was used at 10 μg/ml, anti-PD-1 (Miltenyi Biotec) at 1 μg/ml and anti-PD-L1 (eBioscience, CA, USA) at 1 μg/ml. Nor-NOHA (Calbiochem, Darmstadt, Germany) and 1-MT (Sigma-Aldrich) were added to cultures of activated T cells with i-BM-MDSCs, and Stattic (Calbiochem, Merck Millipore, Darmastadt, Germany) was used at 5 μM to pretreat i-BM-MDSCs for 30 minutes at room temperature, after that cells were washed and added to activated T cells.

After 4 days, cells were harvested, incubated with FcReceptor (FcR) Blocking Reagent (Miltenyi Biotec) and then stained with anti-CD3ε PC7 (Beckman Coulter) antibody. TruCount^TM^ tubes (BD Biosciences) were used to determine the absolute cell number of CD3^+^ cells in the samples. Data acquisition was performed on LSRII ﬂow cytometer (BD Bioscience) and samples were analyzed by FlowJo software (Tree Star, Inc. Ashland, OR, USA). Proliferation of CD3^+^/ CellTrace^+^ T cells was evaluated by assessing the signal of CellTrace on CD3^+^ cells. The extent of T cell proliferation was quantified analyzing the percentage of proliferating cells from generation 3 to generation 10, assumed to be 100% without MDSCs.

To test the immunosuppressive activity of PD-L1^+^ and PD-L1^−^ cells, i-BM-MDSCs were cultured for 20 hours in the presence of CellTrace-labelled anti-CD3/anti-CD28 activated T cells. The culture was harvested and stained with anti-PD-L1 PE (eBioscience) and PD-L1^+^ and PD-L1^−^ cells were separated with FACSAria (Becton Dickinson) by gating myeloid cells on CellTrace^−^ cells. All the fractions were obtained with a purity of at least 95%. The sorted populations were co-coltured for 3 days with CellTrace^+^ T cells pre-activated from 20 hours with anti-CD3/anti-CD28. Suppression was evaluated as described before.

### Flow cytometric analysis, antibodies and reagents

To define the phenotypic changes of myeloid and T cells during the co-culture, cells were stained at different time-points with anti-CD3ε PC7 (Beckman Coulter), anti-PD-L1 PE (BioLegend), anti-PD1 PE (Miltenyi Biotec), anti-LAG-3 FITC (Adipogen, CA, USA). The analysis was performed by gating CD3^−^/CellTrace^−^ or CD3^+^/CellTrace^+^. For staining of PD-L1, PD1 and LAG-3 FMO (Fluorescence Minus One) control was used [43]. To determine the percentage of live, early apoptotic and apoptotic cells at the end of the culture of i-BM-MDSCs with activated T lymphocytes, cells were stained with anti-CD3 PE-Cy7, and subsequently labelled with Annexin V Alexa 647 (BioLegend) plus 7-aminoactinomycin-D (7AAD - eBioscience) for 15 minutes at room temperature and immediately analysed by FACSCalibur.

Cytokine secretion assay for IL-10 was performed following the manufacturer's instructions (Miltenyi Biotec). Briefly, cell cultures of T and myeloid cells were labelled with anti-IL-10 Detection Antibody PE, anti-CD33 APC (BD Bioscience), anti-CD3ε PECy7 (Beckman Coulter) and the signal of IL-10 producing cells was gated on CD33^+^/CD3^−^/CellTrace^−^ or CD3^+^/CellTrace^+^. PBMCs cultured with or without 100 ng/ml of LPS for 14 hours were used as positive and negative control, respectively.

i-BM-MDSCs were analyzed by flow cytometry for the expression of P-STAT3 before and after 20 hours of culture with anti-CD3/CD28 stimulated T cells or control T cells. Cells were fixed with 1% formaldehyde and permeabilized with cold methanol and then stained with anti-human P-STAT3 (Tyr705) mAb (Cell Signaling Technology, MA, USA) and DyLight488-donkey anti-rabbit IgG antibody (BioLegend). The same staining was performed also on PD-L1^+^ and PD-L1^−^ fractions isolated as described above.

### Protein extraction and western blot analysis

Nuclear and cytoplasmic protein fractions were obtained from i-BM-MDSCs and m-BM-MDSCs using NE-PER Nuclear and Cytoplasmic Extraction Reagents (Thermo Scientific, MA, USA). Protein fractions were quantified with Bradford Method and subjected to 10% SDS-PAGE plus electric transfer onto polyvinylidene difluoride membrane (Millipore). Membranes were saturated and incubated with STAT3 and P-STAT3 (Tyr705) mAbs (Cell Signaling Technology), and then with HRP-conjugated donkey anti-rabbit IgG antibody (NA934V, GE Healthcare). Chemioluminescence was developed with SuperSignal West Pico Chemiluminescent Substrate (Thermo Scientific) and signal was acquired by Chemidoc XRS System (Bio-Rad, Hercules, CA, USA). Subsequently, hybridization with mouse anti-human Nucleoporin p62 mAb (BD Transduction Laboratories, CA, USA) and mouse anti-human β-actin mAb (Santa Cruz Biotecnology Inc., TX, USA) followed by a secondary HRP-conjugated, sheep anti-mouse IgG antibody (GE Healthcare).

### ELISA assay

The supernatants of cell cultures of MDSCs and activated T cells were harvested after 4 days of culture ELISA Ready-SET-Go (eBioscience) for IL-10 was performed, following manufacturer's instruction.

### Cell isolation from tumor biopsies

Peripheral blood and biopsies from liver metastases of TNM stage IV colorectal cancer patients and biopsies from melanoma patients were received from the Department of Surgery, Oncology and Gastroenterology of the University of Padova and from the Melanoma Oncology Unit of the Veneto Institute of Oncology (IOV-IRCCS). The project was approved by the Ethics Committee and all patients gave their informed consent. Immediately after the withdrawal, biopsies were dissected and digested in an enzymatic mix composed of 150 mg/l collagenase I, 25 mg/l DNAse I, 150 mg/l collagenase IV, 150 mg/l collagenase II and 25 mg/l elastase, all from Worthington-Biochemical Corporation, NJ, USA. Biopsies were then filtered through a 100 μm cell strainer and labelled with Livedead Aqua (Life Techonologies, MA, USA), anti-CD45 Vioblue (Miltenyi Biotec), anti-CD33 PE-Cy7 (eBioscience) or anti-CD33 APC (BD Bioscience), anti-HLA-DR PerCP-Cy5.5 (BioLegend), Lineage cocktail 1 FITC (BD Bioscience), anti-CD11b Alexa700 (BD Pharmingen), anti-PD-L1 PE (eBioscience), anti-CD14 APC-H7 (BD Bioscience), anti-CD15 FITC (BD Bioscience), anti-CD3 PE-Cy7 (Beckman Coulter), anti-CD8 APC-H7 (BD Bioscience), anti-LAG-3 FITC (AdipoGen), anti-PD1 PE (Miltenyi Biotec). Peripheral blood from healthy donors and patients was saturated and stained with anti-CD3 ECD (Beckman Coulter), anti-CD8 APC-H7 (BD Bioscience), anti-LAG-3 FITC (AdipoGen), anti-PD-1 PE (Miltenyi), red blood cells were lysed by Cal-Lyse Whole Blood Lysing Solution (Life Technologies). After incubation with mAbs, samples were immediately analysed by LSRII flow cytometer. In order to isolate myeloid cells, cell suspension was stained with anti-human CD45 Vioblue (Miltenyi biotec), CD33 PE-Cy7 (eBioscience) and HLA-DR APC (BD Bioscience) and then total CD45^+^/CD33^+^ myeloid cells or cell subsets based on the intensity of HLA-DR and of CD33 on CD45^+^/CD33^+^cells were separated by FACS sorting using MoFlow Astrios (Beckman Coulter). The functional activity of CD33^+^ cell population and of its subsets was tested as previously described. The percentage of suppression was calculated evaluating the ratio between the absolute number of CD3^+^/CellTrace^+^ cells cultured with myeloid cells and the number of the same cells stimulated alone, assuming the proliferation of T cells in the absence of myeloid cells as 100%.

### Real-time RT-PCR analysis

Expression levels of IDO were measured by real-time RT-PCR. Total RNA was used for first-strand cDNA synthesis using the SuperScript™ II Reverse Transcriptase kit and Taqman Assay (Invitrogen by Life Technologies Inc., MA, USA) to detect and quantify IDO mRNA. Relative expression levels were calculated using the comparative Ct method and the fold change was expressed as 2^−ΔΔCt^.

### Statistical analysis

Continuous variables were compared using the Student's *t-*test when data distribution passed the normality test; otherwise the Mann-Whitney U-test was used. Differences were considered statistically significant with *P*<0.05. All statistical analyses were performed using the Sigmaplot software (Systat Software Inc., CA, USA). Absence of significance was not reported for brevity.

## SUPPLEMENTARY MATERIAL FIGURE



## References

[1] Solito S, Marigo I, Pinton L, Damuzzo V, Mandruzzato S, Bronte V (2014). Myeloid-derived suppressor cell heterogeneity in human cancers. Annals of the New York Academy of Sciences.

[2] Marvel D, Gabrilovich DI (2015). Myeloid-derived suppressor cells in the tumor microenvironment: expect the unexpected. The Journal of clinical investigation.

[3] Marigo I, Bosio E, Solito S, Mesa C, Fernandez A, Dolcetti L, Ugel S, Sonda N, Bicciato S, Falisi E, Calabrese F, Basso G, Zanovello P, Cozzi E, Mandruzzato S, Bronte V (2010). Tumor-induced tolerance and immune suppression depend on the C/EBPbeta transcription factor. Immunity.

[4] Solito S, Falisi E, Diaz-Montero CM, Doni A, Pinton L, Rosato A, Francescato S, Basso G, Zanovello P, Onicescu G, Garrett-Mayer E, Montero AJ, Bronte V, Mandruzzato S (2011). A human promyelocytic-like population is responsible for the immune suppression mediated by myeloid-derived suppressor cells. Blood.

[5] Gabrilovich DI, Nagaraj S (2009). Myeloid-derived suppressor cells as regulators of the immune system. Nature reviews Immunology.

[6] Sippel TR, White J, Nag K, Tsvankin V, Klaassen M, Kleinschmidt-DeMasters BK, Waziri A (2011). Neutrophil degranulation and immunosuppression in patients with GBM: restoration of cellular immune function by targeting arginase I. Clinical cancer research.

[7] Srivastava MK, Sinha P, Clements VK, Rodriguez P, Ostrand-Rosenberg S (2010). Myeloid-derived suppressor cells inhibit T-cell activation by depleting cystine and cysteine. Cancer research.

[8] Bronte V, Zanovello P (2005). Regulation of immune responses by L-arginine metabolism. Nature reviews Immunology.

[9] Chou HS, Hsieh CC, Charles R, Wang L, Wagner T, Fung JJ, Qian S, Lu LL (2012). Myeloid-derived suppressor cells protect islet transplants by B7-H1 mediated enhancement of T regulatory cells. Transplantation.

[10] Duraiswamy J, Freeman GJ, Coukos G (2013). Therapeutic PD-1 pathway blockade augments with other modalities of immunotherapy T-cell function to prevent immune decline in ovarian cancer. Cancer research.

[11] Fujimura T, Ring S, Umansky V, Mahnke K, Enk AH (2012). Regulatory T cells stimulate B7-H1 expression in myeloid-derived suppressor cells in ret melanomas. The Journal of investigative dermatology.

[12] Liu Y, Zeng B, Zhang Z, Zhang Y, Yang R (2008). B7-H1 on myeloid-derived suppressor cells in immune suppression by a mouse model of ovarian cancer. Clin Immunol.

[13] Zhang B, Wang Z, Wu L, Zhang M, Li W, Ding J, Zhu J, Wei H, Zhao K (2013). Circulating and tumor-infiltrating myeloid-derived suppressor cells in patients with colorectal carcinoma. PloS one.

[14] Rebe C, Vegran F, Berger H, Ghiringhelli F (2013). STAT3 activation: A key factor in tumor immunoescape. Jak-Stat.

[15] Nefedova Y, Nagaraj S, Rosenbauer A, Muro-Cacho C, Sebti SM, Gabrilovich DI (2005). Regulation of dendritic cell differentiation and antitumor immune response in cancer by pharmacologic-selective inhibition of the janus-activated kinase 2/signal transducers and activators of transcription 3 pathway. Cancer research.

[16] Darnell JE, Kerr IM, Stark GR (1994). Jak-STAT pathways and transcriptional activation in response to IFNs and other extracellular signaling proteins. Science.

[17] Cheng P, Corzo CA, Luetteke N, Yu B, Nagaraj S, Bui MM, Ortiz M, Nacken W, Sorg C, Vogl T, Roth J, Gabrilovich DI (2008). Inhibition of dendritic cell differentiation and accumulation of myeloid-derived suppressor cells in cancer is regulated by S100A9 protein. The Journal of experimental medicine.

[18] Vasquez-Dunddel D, Pan F, Zeng Q, Gorbounov M, Albesiano E, Fu J, Blosser RL, Tam AJ, Bruno T, Zhang H, Pardoll D, Kim Y (2013). STAT3 regulates arginase-I in myeloid-derived suppressor cells from cancer patients. The Journal of clinical investigation.

[19] Huard B, Prigent P, Tournier M, Bruniquel D, Triebel F (1995). CD4/major histocompatibility complex class II interaction analyzed with CD4- and lymphocyte activation gene-3 (LAG-3)-Ig fusion proteins. European journal of immunology.

[20] Liang B, Workman C, Lee J, Chew C, Dale BM, Colonna L, Flores M, Li N, Schweighoffer E, Greenberg S, Tybulewicz V, Vignali D, Clynes R (2008). Regulatory T cells inhibit dendritic cells by lymphocyte activation gene-3 engagement of MHC class II. J Immunol.

[21] Blackburn SD, Shin H, Haining WN, Zou T, Workman CJ, Polley A, Betts MR, Freeman GJ, Vignali DA, Wherry EJ (2009). Coregulation of CD8+ T cell exhaustion by multiple inhibitory receptors during chronic viral infection. Nature immunology.

[22] Butler NS, Moebius J, Pewe LL, Traore B, Doumbo OK, Tygrett LT, Waldschmidt TJ, Crompton PD, Harty JT (2012). Therapeutic blockade of PD-L1 and LAG-3 rapidly clears established blood-stage Plasmodium infection. Nature immunology.

[23] Grosso JF, Goldberg MV, Getnet D, Bruno TC, Yen HR, Pyle KJ, Hipkiss E, Vignali DA, Pardoll DM, Drake CG (2009). Functionally distinct LAG-3 and PD-1 subsets on activated and chronically stimulated CD8 T cells. J Immunol.

[24] Wherry EJ (2011). T cell exhaustion. Nature immunology.

[25] Williams L, Bradley L, Smith A, Foxwell B (2004). Signal transducer and activator of transcription 3 is the dominant mediator of the anti-inflammatory effects of IL-10 in human macrophages. J Immunol.

[26] Marzec M, Zhang Q, Goradia A, Raghunath PN, Liu X, Paessler M, Wang HY, Wysocka M, Cheng M, Ruggeri BA, Wasik MA (2008). Oncogenic kinase NPM/ALK induces through STAT3 expression of immunosuppressive protein CD274 (PD-L1, B7-H1). Proceedings of the National Academy of Sciences of the United States of America.

[27] Dibra D, Li S (2013). The cell-to-cell coordination between activated T cells and CpG-stimulated macrophages synergistically induce elevated levels of IL-10 via NF-kappaB1, STAT3, and CD40/CD154. Cell communication and signaling : CCS.

[28] Aggarwal BB, Kunnumakkara AB, Harikumar KB, Gupta SR, Tharakan ST, Koca C, Dey S, Sung B (2009). Signal transducer and activator of transcription-3, inflammation, and cancer: how intimate is the relationship?. Annals of the New York Academy of Sciences.

[29] Panopoulos AD, Zhang L, Snow JW, Jones DM, Smith AM, El Kasmi KC, Liu F, Goldsmith MA, Link DC, Murray PJ, Watowich SS (2006). STAT3 governs distinct pathways in emergency granulopoiesis and mature neutrophils. Blood.

[30] Zhang H, Nguyen-Jackson H, Panopoulos AD, Li HS, Murray PJ, Watowich SS (2010). STAT3 controls myeloid progenitor growth during emergency granulopoiesis. Blood.

[31] Abad C, Nobuta H, Li J, Kasai A, Yong WH, Waschek JA (2014). Targeted STAT3 disruption in myeloid cells alters immunosuppressor cell abundance in a murine model of spontaneous medulloblastoma. Journal of leukocyte biology.

[32] Feng PH, Lee KY, Chang YL, Chan YF, Kuo LW, Lin TY, Chung FT, Kuo CS, Yu CT, Lin SM, Wang CH, Chou CL, Huang CD, Kuo HP (2012). CD14(+)S100A9(+) monocytic myeloid-derived suppressor cells and their clinical relevance in non-small cell lung cancer. American journal of respiratory and critical care medicine.

[33] Said EA, Dupuy FP, Trautmann L, Zhang Y, Shi Y, El-Far M, Hill BJ, Noto A, Ancuta P, Peretz Y, Fonseca SG, Van Grevenynghe J, Boulassel MR, Bruneau J, Shoukry NH, Routy JP (2010). Programmed death-1-induced interleukin-10 production by monocytes impairs CD4+ T cell activation during HIV infection. Nature medicine.

[34] Rodriguez-Garcia M, Porichis F, de Jong OG, Levi K, Diefenbach TJ, Lifson JD, Freeman GJ, Walker BD, Kaufmann DE, Kavanagh DG (2011). Expression of PD-L1 and PD-L2 on human macrophages is up-regulated by HIV-1 and differentially modulated by IL-10. Journal of leukocyte biology.

[35] Wolfle SJ, Strebovsky J, Bartz H, Sahr A, Arnold C, Kaiser C, Dalpke AH, Heeg K (2011). PD-L1 expression on tolerogenic APCs is controlled by STAT-3. European journal of immunology.

[36] Zoso A, Mazza EM, Bicciato S, Mandruzzato S, Bronte V, Serafini P, Inverardi L (2014). Human fibrocytic myeloid-derived suppressor cells express IDO and promote tolerance via Treg-cell expansion. European journal of immunology.

[37] Yu J, Du W, Yan F, Wang Y, Li H, Cao S, Yu W, Shen C, Liu J, Ren X (2013). Myeloid-derived suppressor cells suppress antitumor immune responses through IDO expression and correlate with lymph node metastasis in patients with breast cancer. J Immunol.

[38] Macon-Lemaitre L, Triebel F (2005). The negative regulatory function of the lymphocyte-activation gene-3 co-receptor (CD223) on human T cells. Immunology.

[39] Nagaraj S, Nelson A, Youn JI, Cheng P, Quiceno D, Gabrilovich DI (2012). Antigen-specific CD4(+) T cells regulate function of myeloid-derived suppressor cells in cancer via retrograde MHC class II signaling. Cancer research.

[40] Matsuzaki J, Gnjatic S, Mhawech-Fauceglia P, Beck A, Miller A, Tsuji T, Eppolito C, Qian F, Lele S, Shrikant P, Old LJ, Odunsi K (2010). Tumor-infiltrating NY-ESO-1-specific CD8+ T cells are negatively regulated by LAG-3 and PD-1 in human ovarian cancer. Proceedings of the National Academy of Sciences of the United States of America.

[41] Woo SR, Turnis ME, Goldberg MV, Bankoti J, Selby M, Nirschl CJ, Bettini ML, Gravano DM, Vogel P, Liu CL, Tangsombatvisit S, Grosso JF, Netto G, Smeltzer MP, Chaux A, Utz PJ (2012). Immune inhibitory molecules LAG-3 and PD-1 synergistically regulate T-cell function to promote tumoral immune escape. Cancer research.

[42] Mandruzzato S, Solito S, Falisi E, Francescato S, Chiarion-Sileni V, Mocellin S, Zanon A, Rossi CR, Nitti D, Bronte V, Zanovello P (2009). IL4Ralpha+ myeloid-derived suppressor cell expansion in cancer patients. J Immunol.

[43] Perfetto SP, Chattopadhyay PK, Roederer M (2004). Seventeen-colour flow cytometry: unravelling the immune system. Nature reviews Immunology.

