# Supplementary material for: Activated T cells sustain myeloid-derived suppressor cell-mediated immune suppression
Source: Oncotarget. 2015 Dec 18;7(2):1168–84. doi: 10.18632/oncotarget.6662 (PMC4811451; doi:10.18632/oncotarget.6662)
Supplement: Supplementary file 1 [file oncotarget-07-1168-s001.pdf]

Activated T cells sustain myeloid-derived suppressor cell-mediated immune suppression

Supplementary Material

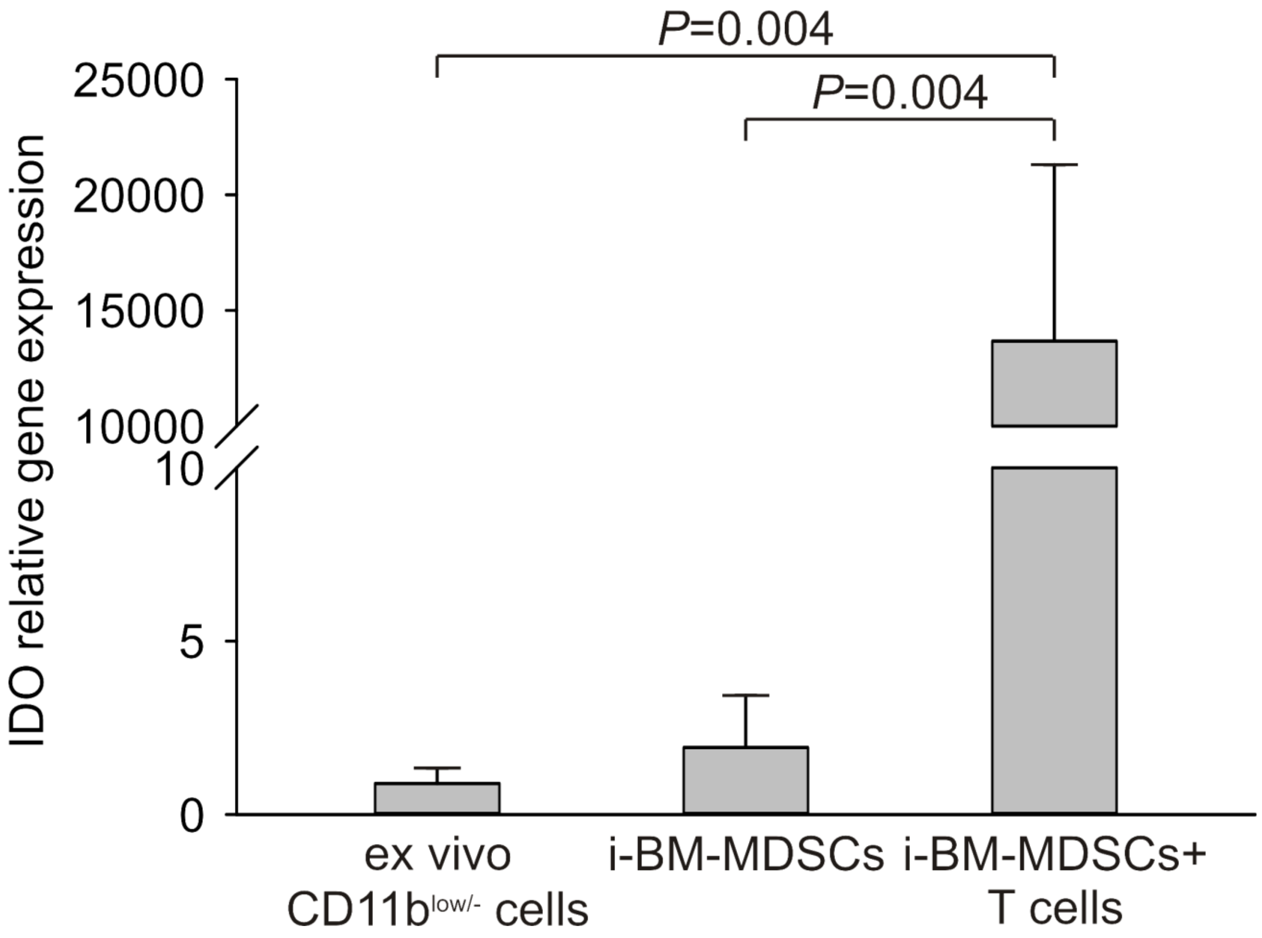

Supplementary Figure 1: **IDO expression on i-BM-MDSCs.** IDO expression levels were evaluated by real-time RT-PCR on CD11b$^{low/-}$ BM cells isolated *ex vivo*, on i-BM-MDSCs and on the same cells isolated after 20 hours of co-culture with activated T cells. Mann-Whitney U test was applied.
